# Salty, Sweet and Difficult to Treat: A Case of Profound Hypernatremia in the Setting of Hyperosmotic Hyperglycemic State

**DOI:** 10.7759/cureus.7278

**Published:** 2020-03-15

**Authors:** Raman J Sohal, Sumendra Joshi

**Affiliations:** 1 Medicine, State University of New York (SUNY) Upstate Medical University, Syracuse, USA; 2 Pulmonology and Critical Care, State University of New York (SUNY) Upstate Medical University Hospital, Syracuse, USA

**Keywords:** hypernatremia, hhs, altered mental status, confusion, hyperglycemia, rhabdomyolysis, sepsis

## Abstract

Hyperosmolar hyperglycemic state (HHS) is a disorder that occurs most frequently in type 2 diabetics and is associated with high mortality - up to 50%. Hypernatremia, when associated with HHS, worsens the prognosis. Encephalopathy is evident at a serum sodium level greater than 160 mOsm/kg. Additional symptoms include lethargy, weakness, seizures, and coma. Rhabdomyolysis can rarely occur in hyperosmolar states. Here we describe a case of severe hypernatremia in the setting of HHS leading to profound encephalopathy and report to the best of our knowledge the highest serum sodium level published in the literature. A 50-year-old female with no past medical history (PMH) of diabetes presented to the ED obtunded and found to have a glucose level of 1400 mg/dL without metabolic acidosis or ketosis. Her sodium on presentation was 169 mOsm/kg but subsequently rose to 200 mOsm/kg when corrected for hyperglycemia. Plasma osmolality was 340 mOsm/kg. She developed pre-renal acute kidney injury (AKI) secondary to the osmotic diuresis from severe hyperglycemia as well as rhabdomyolysis with a peak creatine kinase(CK) level of 2493. The free water deficit was 14L which was corrected. New-onset anisocoria raised concern for osmotic demyelination which was further investigated with MRI. An acute ischemic stroke in the right caudate was found. Fortunately, the patient survived the endocrine emergency. This case emphasizes the importance of an appropriate rate of sodium correction. This case is particularly unique because the degree of hypernatremia seen here was in the absence of intentional salt loading (for example by the administration of hypertonic saline), or psychiatric disease (as psychogenic adipsia). In conclusion, we report the case of severe hypernatremia and the highest documented serum sodium level was seen in literature in the background of HHS, rhabdomyolysis and septic shock.

## Introduction

Hyperosmolar hyperglycemic state (HHS) is an endocrinopathy of high mortality with a rate between 10-50%. It is usually precipitated by infection or stressor and maybe the first presentation of diabetes mellitus. HHS frequently occurs in type 2 diabetes mellitus with the serum osmolality reaching greater than 340 mOsm/kg and blood glucose levels greater than 600 mg/dL although they are frequently higher [1,2]. Altered mental status is the most frequent presentation. Advanced age, a known source of precipitation, the degree of hyper-osmolality, low Glasgow coma scale (GCS) score, and hemodynamic instability are frequent factors that predispose to a poorer prognosis [3]. Rhabdomyolysis is a less reported complication that can be seen in hyperosmolar syndromes including in cases of hypernatremia and hyperglycemia [4]. Here we report a case of severe hypernatremia secondary to HHS that was complicated by the development of septic shock, stroke, and rhabdomyolysis. This case seeks to report to the best of our knowledge one of the highest documented sodium levels in the absence of intentional salt loading or ingestion [5].

## Case presentation

**Figure 1:**
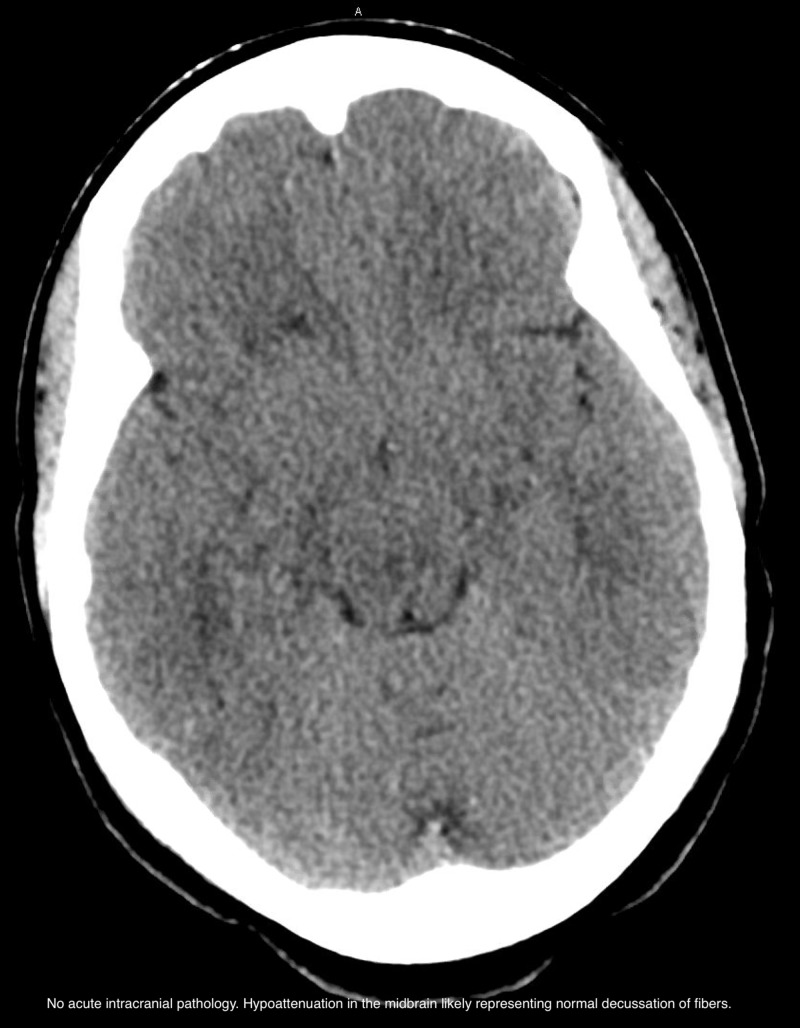
CT head non-contrast

**Figure 2:**
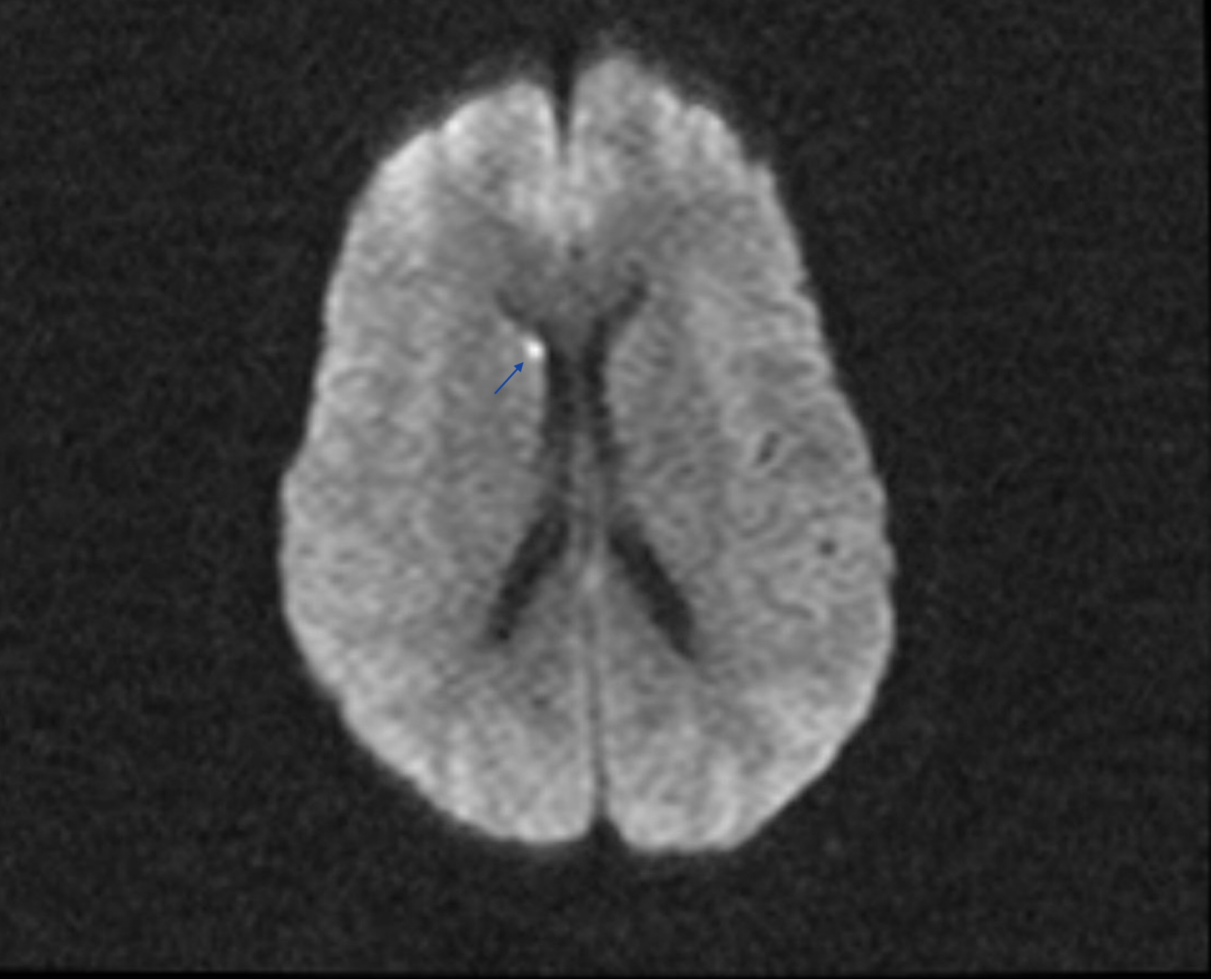
MRI brain with contrast Blue arrow points to the area of acute infarct.

**Figure 3:**
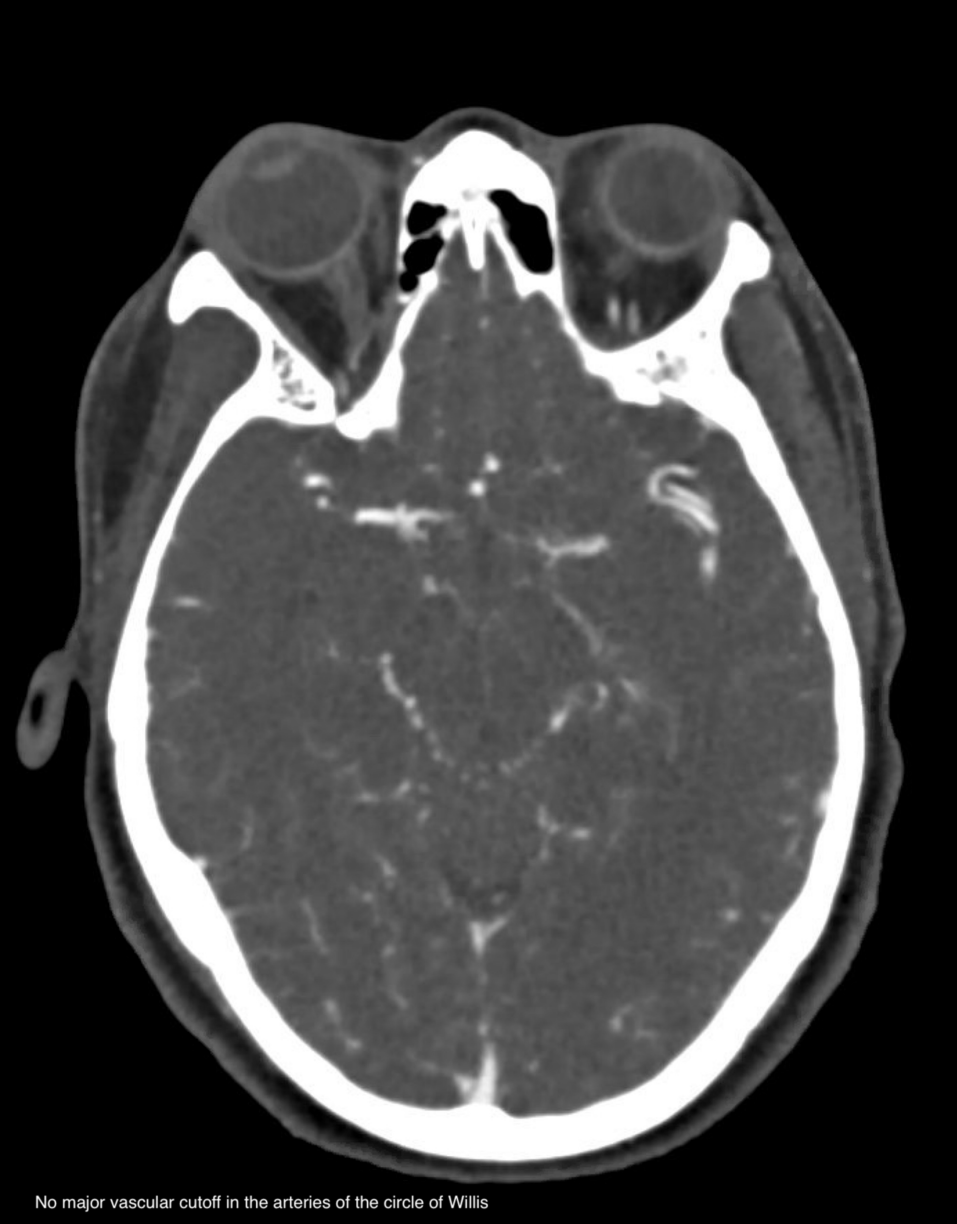
Computed tomography angiography (CTA) head and neck

A 50-year-old otherwise healthy female was brought to the ED for confusion. Leading up the hospitalization the patient had developed progressive lethargy, confusion, and altered gait. On presentation glucose level was 1400, with no known history of diabetes mellitus, and her sodium was 169 which subsequently rose greater than 180 (upper limit for our lab is 180) within the next few hours. The corrected sodium level was greater than 200 mmol/L. The daily trend of the serum sodium, serum glucose, and corrected sodium level is shown in Table 1. Her total serum osmolality was 437 mOsm/kg. Anion gap and serum bicarbonate were within normal limits. The triad of severe hyperglycemia and altered mentation in the absence of metabolic acidosis or ketosis established the diagnosis of the hyperosmotic hyperglycemic syndrome (HHS). Her HbA1c was greater than 14. In this case, the patient was not known to have prior diabetes. Her last HbA1C three years prior was 5.8. She did have E. faecalis urinary tract infection (UTI) and septic shock which was the likely precipitant of the HHS. Her course was complicated by rhabdomyolysis with contributing intrinsic renal injury. CK level at peak reached 2493. She was intubated and mechanically ventilated for encephalopathy. She had a 14 L free water deficit. It was slowly corrected with the goal to bring the sodium down to 180 and then to decrease by 10 mEq every 24 hrs. Following initial volume replacement with isotonic normal saline, D5 water was used to correct the sodium. HHS was treated with insulin glucose tolerance test (GTT). Her GAD-65 and ZNT8 auto antibodies sent for type 1 diabetes workup were negative. She developed aspiration pneumonia and septic shock and was treated with antibiotics and norepinephrine. On day 4, anisocoria was noted on physical exam and CT head non-contrast showed hypoattenuation in the midbrain concerning for osmotic demyelination syndrome given the rapid correction of sodium from 200 to 160 over three days (Figure 1). However, the final report stated that this may be likely representing the normal decussation of the pontine fibers and that follow-up with MRI was recommended. MRI revealed an acute right caudate infarct but there was no evidence of pontine myelinolysis (Figure 2). Workup of stroke including computed tomography angiography (CTA) head and neck (Figure 3), telemetry and echo with bubble study was unrevealing. Sodium was corrected over six days. Encephalopathy resolved following resolution of hypernatremia, HHS and septic shock and she was subsequently extubated and made an uneventful recovery. She was discharged on day 13 of hospitalization. Unfortunately, she has been lost to follow up.

**Table 1:** Serum sodium and glucose laboratory level with corrected sodium level for hyperglycemia Glucose reference range: 70-140 mg/dL Sodium reference range: 136-145 mmol/L

Date	Laboratory Sodium Level (mmol/L)	Serum Glucose (mg/dL)	Corrected Sodium Level Hillier, 1999 (mmol/L)
Day 1	169	1394	200
	173	756	189
	>180	537	190
Day 2	>180	391	190
Day 3	176	303	181
	168	259	172
	172	149	173
	167	253	171
	162	307	167
Day 4	159	282	163
	150	252	154
Day 5	155	296	169
	153	165	155
Day 6	151	187	155
	146	272	149
	142	200	144
Day 7	147	157	148
	144	136	145
Day 8	142	163	144
Day 9	141	251	145
Day 10	143	194	145
Day 11	143	117	143
Day 12	140	95	140

## Discussion

Review of the literature reveals that cases of severe hypernatremia have been secondary to either intentional toxic salt load ingestion or the inappropriate administration/preparation of hypertonic saline/dialysate, respectively [5]. To the best of our knowledge, we report the highest case of severe hypernatremia with corrected sodium measuring 200 that was not secondary to unintentional/erroneous salt loading. Encephalopathy is evident at sodium levels of greater than 160, with a total plasma osmolality of >340 [2,4]. Whether hypo- or hypernatremia develops in a case of HHS is dependent on the relative balance of the more prominent factor: increased plasma osmolality driving free water into the extracellular space leading to dilutional hyponatremia versus the loss of that free water through osmotic diuresis leading to free water deficit and hypovolemia with true hypernatremia. In our case, the degree of hyperglycemia and hyperosmolality had tipped in favor of strong diuresis leading to a free water deficit of 14 L and profound hypernatremia. The average water deficit in HHS is 9 L, and our patient had a 150% higher water deficit again exemplifying the severity of this hypernatremia. This to the best of our knowledge is the highest reported level of hypernatremia even compared in face of intentional salt loading which has been frequently reported causes of severe hypernatremia. The degree of intravascular volume depletion was so severe as to lead to pre-renal AKI and tissue hypo-perfusion in rhabdomyolysis. Such an event is rare in cases of hypernatremia and again demonstrates the severe degree of hypernatremia.

In this case, we report hypernatremia of 200 and hyperosmolality of 437 secondary to HHS with admission glucose of 1394. Poor outcomes are noted in patients with advanced age (using cut off of 65 years), the degree of hyperosmolality, hemodynamic compromise, low GCS score on presentation and a known source of infection [3]. Our patient met four out of the five criteria, making her prognosis dismal, however, she recovered successfully. The cornerstone of therapy for HHS and hypernatremia, in this case, is vigorous hydration but care to be noted in the rate of decrease of the serum sodium. In our patient, the corrected sodium decreased from 200 to the mid 160s within three days. The initial anisocoria prompted concern for osmotic demyelination syndrome (ODS), however, MRI was not suggestive but did reveal a new acute infarct in the right caudate. The etiology is unclear for why. Again, this case stresses the importance of appropriate sodium correction in patients with hypernatremia especially when there is ongoing hyperglycemia. Endocrinopathy mediated rhabdomyolysis is a rare and much less reported consequence of HHS or hypernatremia [4]. Singhal et al. first described determinants of rhabdomyolysis which included increased serum osmolality [6,7]. In our case, we have two contributing factors: hyperglycemia and hypernatremia. This is significant because this can be a cause of AKI that is frequently overlooked by the larger metabolic picture and kidney function may continue to deteriorate despite fluid resuscitation. The exact mechanism of rhabdomyolysis is unclear but likely related to the inhibition of the sodium-calcium pump leading to increased cytoplasmic calcium concentration [4]. Here we report rhabdomyolysis as less common and even less reported complication of HHS and hypernatremia. Our patient presented with a creatinine level of 2.22, with a baseline of 0.5. Her CK level was elevated at 2493, which subsequently improved to 1163 over three days with vigorous hydration.

## Conclusions

Hypernatremia secondary to HHS is a combination of high mortality incidence. Here two major factors could have led to the unrelenting encephalopathy including the hyperglycemia and the hypernatremia, which was further confounded by the presence of the ongoing infection. Rhabdomyolysis can be a complication of high plasma osmolality. We report to the best of our knowledge one of the highest serum sodium levels in the absence of intentional salt ingestion or psychiatric disease.
